# The Cigar Factory “Reader”

**Published:** 1910-05

**Authors:** 


### The Cigar Factory "Reader."

An exceedingly interesting feature of the cigar factories is the "reader." The cigar makers are very quiet in the workrooms, and from a favorable position a man termed, the "reader" reads aloud to them from the newspapers or from some novel while they are at work. To a person on the outlook for public-health agencies, the thought immediately occurs that in this "reader" may be found a person who can be of considerable service for good. If he were supplied with printed public-health material, written in popular style, he could, by reading this to the cigar makers, disseminate a great deal of useful information to a large number of people who, themselves, do comparatively little reading.—C. W. STILES, *in Public Health Reports.*

---

Shallow, catching, irregular breathing is characteristic of diaphragmatic inflammation—either peritoneal or pleural.—*American Journal of Surgery.*

